# The Report of the Board of Health, in Manchester, at the Eleventh Annual Meeting, June 1, 1806

**Published:** 1806-10

**Authors:** 


### The Report of the Board of Health, *in Manchester, at the Eleventh Annual Meeting, June* 1, 1806.

THIS institution, which in its infancy had to contend with many difficulties, has now attained such a period of maturity, as in future to secure it against a recurrence of similar obstacles. Its utility has long been placed beyond the reach of controversy, and its importance to the welfare of the inhabitants of this town, is now established upon the solid and indisputable basis of experience.

To meliorate the condition of the lower classes of society, and lighten the pressure of those calamities which are the necessary attendants on poverty, must ever be regarded as the principal ends of a rational philanthropy. But, though involving the highest interests of the community, and calling loudly for our most active exertions, they are objects confessedly the most arduous and most difficult of attainment. The friends of humanity have too often found their wishes disappointed, and their intentions frustrated, by imperfections in the plan, or abuses in the management of public institutions. From this general censure, indeed, the present House of Recovery claims an honourable exemption. There is, perhaps, no charitable institution which has met with such entire success, or more completely realized the hopes of its projectors: there is certainly tainly none which reflects more credit on the liberality and public spirit of the town to which it owes its existence.

As it may afford satisfaction to the public, we take this opportunity of stating, although, at the present period, it would be superfluous to dwell upon the subject, that in every instance in which patients have been received into the house, the diffusion of febrile contagion has been completely prevented; nor has a single instance occurred, in which the most distant suspicion of the communication of the disease after admission, could possibly be entertained.

But while there is happily no longer occasion to correct former misconceptions, it may, perhaps, be not unseasonable to advert to some circumstances connected with the charity, which, we conceive, may, in future, affect its interests. It is the fate of many similar institutions, that in the progress of continued success, the causes from which that success originally proceeded, are overlooked, and at length forgotten. The permanence of the benefits resulting from them, has a tendency to render those benefits less conspicuous, because not so immediately contrasted with the evils they have removed. Those who are unacquainted with the history of the present establishment, are not capable of duly appreciating all the advantages that have accrued from it. We no longer witness the dreadful effects of those widely diffused epidemics, which formerly raged in this town and its neighbourhood, and by which such misery was often entailed, even upon those who were not their immediate victims. As the object of the charity is as much the prevention of sickness among those yet in health, as the relief of those actually sick, the extent of the benefits conferred is liable to be imperfectly estimated, and still less adequately acknowledged. Measures which prevent evil, in proportion to their efficacy, operate secretly and silently. At the very time the benefits are publicly and extensively felt, it is seldom that the bulk of mankind are disposed to exert sufficient reflection to trace them to their real source, or exercise sufficient candour to ascribe the due proportion of merit to their authors.

So completely have the ends originally proposed by the Board of Health, been answered by the present institution, that ever since the new building has been opened, the number of patients requiring admission has been short of what was expected: the average has indeed borne a small proportion to the number the house was capable of accommodating. A more convincing argument of the efficacy of the plans adopted for the extinction of contagion could hardly be adduced. To expect that the building might ever become entirely useless, would be to indulge, perhaps, too sanguine a hope. For as long as the poor remain so regardless of their real interests, as to lavish the produce of their industry in the purchase of baneful and enervating luxuries, and, by living in confined and crowded habitations, are willing to forego the comforts of cleanliness, pure air, and wholesome diet, for the temporary gratifications of perverted appetite, so long will dangerous fevers arise among them, so long will their abodes be fruitful sources of infection, and so long will their constitutions readily receive and quickly generate the poisonous exhalations of typhus.

It is however extremely pleasing to contemplate the progress that has already been made in our own town: and we may reasonably hope that the gradual increase of knowledge, and consequent improvement of manners, will operate still more beneficial changes. As by the measures adopted by our predecessors, we are now relieved from the terrors of the plague, and other distempers, which formerly spread desolation throughout Europe; let us hope that still further improvements are reserved for succeeding times. The recent discovery, which has divested of every danger, and promises to extirpate the small-pox, will, doubtless, rank among the most important benefits which science has conferred upon mankind. Nor with respect to fever, is it too much to presume, that means may be found, which by disarming contagion of its virulence, may reduce the chances of infection, and diminish the uncertainty and danger attendant on every form of the disease. Discoveries of improved methods of cure, are, it is obvious, more likely to result from the reception of patients into hospitals, where the progress of diseases is more open to observation, and where, from their being placed in similar circumstances, the effects of different modes of treatment can be more accurately ascertained. In the wards of an hospital, also, a better and more judicious attendance is bestowed upon the patients, than they could possibly receive at their own houses, where they are so likely to be injured from the ignorant zeal, and officious interference of their friends. This circumstance alone might be sufficient to account for the acknowledged fact, that "More patients recover of fevers in hospitals than in private families, under similar practice."* During

* Fordyce: Transactions of a Society, &c. page 12.

During the last year, the proportion of deaths among the patients in the house has not been so great as the preceding year: but as during some former years it has been even less than at present, it may be proper to make a few observations upon this apparent diminution in the success of the practice.

The average proportion of mortality during the last year, has been one in nine. When it is considered that in hospitals, which admit a variety of diseases, both chronic and acute, the general proportion is one in twelve, this augmentation, in an hospital destined for the reception of the worst cases of fever, will appear nowise surprising. But, as during the first years of the establishment of the Fever-wards, the number of deaths was not more than one in eleven, it may be expedient to state some farther circumstances which account for the variation.

The danger attending fevers depends much on the nature of the season, and of the prevailing epidemics. With an equal exertion of skill and attention on the part of the physicians, the result of their practice will be very different in different years. This will appear from the following table, exhibiting the proportion between the deaths and recoveries at different periods since the house has been opened.

(*The house was opened in May,* 1796.)

| Years. | THE NUMBER OF PATIENTS. | | | | Proportion of Deaths. |
|---|---|---|---|---|---|
| | Admitted. | Cured. | Dead. | Remaining at the end of each year. | |
| From 1796 to 1797 | 371 | 324 | 40 | 7 | 1 in 9 |
| 1797 — 1798 | 339 | 300 | 16 | 23 | 1 — 20 |
| 1798 — 1799 | 393 | 360 | 27 | 11 | 1 — 14 |
| 1799 — 1800 | 364 | 315 | 41 | 8 | 1 — 9 |
| 1800 — 1801 | 747 | 645 | 63 | 39 | 1 — 11 |
| 1801 — 1802 | 1070 | 956 | 84 | 30 | 1 — 12 |
| 1802 — 1803 | 601 | 539 | 53 | 9 | 1 — 11 |
| 1803 — 1804 | 256 | 215 | 33 | 8 | 1 — 7 |
| 1804 — 1805 | 184 | 144 | 34 | 6 | 1 — 5¼ |
| 1805 — 1806 | 268 | 235 | 29 | 4 | 1 — 9 |
| Total | 4453 | 4033 | 420 | | |

 From

From 1804 to 1805, many cases were admitted of a most lingering and dangerous kind. Hardly any patient brought into the house escaped without hazard, and many deaths took place from sudden changes in the state of the fever, contrary to the usual course of the disease, and only imputable to the peculiar character of the epidemic. Similar cases occurred at that time in private practice.

Since the opening of the New House of Recovery, the patients admitted have been chiefly selected by the physicians themselves: the clerk having only power to admit those who reside beyond the limits of the districts. Slight cases are therefore scrupulously rejected; and patients are received only in that state in which the fever is either dangerous to themselves, or liable to be communicated to others. The New House of Recovery has consequently contained a greater number of hazardous cases than was to be found, during any equal period, in the buildings originally occupied for the same purpose. But the ends of the institution have thus been more fully answered. The progress of infectious fever has been effectually arrested; and the destructive epidemic of scarlet fever, which was actually introduced into the town during 1805, from Liverpool, has been completely suppressed. In effecting these purposes, so important to society, so consoling to humanity, the physicians have regarded the public good more than their own immediate reputation; and have preferred the solid benefit of preventing the wide diffusion of contagion, to an ostentatious list of cures, which might easily have been swelled to any amount, by the admission of cases not conducive to the views of the subscribers, or to the welfare of society.

Desirous as the Board must be to second these disinterested proceedings of the Faculty, and anxiously as they must wish to discountenance every ungenerous reflection on the event of their practice, they feel it a duty incumbent upon them to notice a peculiar hardship, to which the physicians of the House of Recovery are frequently exposed, and in the redress of which the public are deeply interested. By the rules of this institution, the servants of private families, who are seized with contagious fever, may be received into the house on certain conditions. The application for the removal of patients of this class is often delayed till the approach of death is apprehended: the odium of the fatal event is thus transferred to a public institution. This evil has been long a subject of complaint and remonstrance; it might easily be remedied by an an early application to the House of Recovery; and common observation is sufficient to indicate when the existence of fever is to be apprehended.

It may likewise be proper to remark a circumstance, which, though sufficiently obvious, has not been generally taken into account: that, when the average number of patients is small, a slight increase of mortality appears alarming, which, in a more crowded state of the house, would excite little notice. During the prevalence of former epidemics, many patients have been thrown into the house, whose cases would not be deemed admissible, at a period when more deliberation could be employed. Others have been received as members of an infected family, with a view of clearing out the house, and giving an opportunity of thoroughly cleansing it.

But it is not by the comparative number of cures, that the success of this charity is to be estimated. It is in the scanty number of fever-patients in the town, and the reduced lists of home-patients of the Infirmary, notwithstanding the increase of population, and the embarrassments of commerce, that we trace the good effects of the institution. While we can assert with confidence, that we possess, by its means, the power of checking one of the great scourges of mankind, we rest assured of the cordial support, and zealous co-operation of every true friend to humanity.

### Officers elected for the Year ensuing.

Sir Robert Peel, Bart. M. P. President.
Mr. Robert Spear, Vice-President.
Mr. Nathaniel Gould, Treasurer.
Mr. Richard Meadowcroft, House Visitor.
Dauntesey Hulme, Esq. & John Drinkwater, Esq. Auditors.

**Physicians.**

John Ferriar, M. D.

S. A. Bardsley, M. D.

Edward Holme, M. D.

Peter Roget, M. D.

Henry Dewar, M. D.

John Underhill, M. D.

**Surgeons.**

Mr. William Simmons.
Mr. Gavin Hamilton.
Mr. Benjamin Gibson.
Mr. John Thorpe.
Mr. John Ransome.

Mr. Godfrey Fox, Jun. Resident Clerk.
Mrs. E. Rutherford, Matron.

